# Systematic characterization of immunoglobulin loci and deep sequencing of the expressed repertoire in the Atlantic cod (*Gadus morhua*)

**DOI:** 10.1186/s12864-024-10571-0

**Published:** 2024-07-03

**Authors:** Ádám Györkei, Finn-Eirik Johansen, Shuo-Wang Qiao

**Affiliations:** 1 Department of Biosciences, Section for Physiology and Cell Biology, University of Oslo, Oslo, Norway; 2 Department of Immunology, Institute of Clinical Medicine, University of Oslo, Oslo, Norway

**Keywords:** Atlantic cod, Immunogenetics, Ig, Repertoire, Diversity, Gadmor3.0

## Abstract

**Background:**

The Atlantic cod is a prolific species in the Atlantic, despite its inconsistent specific antibody response. It presents a peculiar case within vertebrate immunology due to its distinct immune system, characterized by the absence of MHCII antigen presentation pathway, required for T cell-dependent antibody responses. Thorough characterisation of immunoglobulin loci and analysis of the antibody repertoire is necessary to further our understanding of the Atlantic cod’s immune response on a molecular level.

**Results:**

A comprehensive search of the cod genome (gadmor3.0) identified the complete set of IgH genes organized into three sequential translocons on chromosome 2, while IgL genes were located on chromosomes 2 and 5. The Atlantic cod displayed a moderate germline V gene diversity, comprising four V gene families for both IgH and IgL, each with distinct chromosomal locations and organizational structures. 5’RACE sequencing revealed a diverse range of heavy chain CDR3 sequences and relatively limited CDR3 diversity in light chains. The analysis highlighted a differential impact of V-gene germline CDR3 length on receptor CDR3 length between heavy and light chains, underlining different recombination processes.

**Conclusions:**

This study reveals that the Atlantic cod, despite its inconsistent antibody response, maintains a level of immunoglobulin diversity comparable to other fish species. The findings suggest that the extensive recent duplications of kappa light chain genes do not result in increased repertoire diversity. This research provides a comprehensive view of the Atlantic cod's immunoglobulin gene organization and repertoire, necessary for future studies of antibody responses at the molecular level.

**Supplementary Information:**

The online version contains supplementary material available at 10.1186/s12864-024-10571-0.

## Introduction

A fundamental characteristic of the adaptive immune system of jawed vertebrates is the antigen-specific antibody response. This sophisticated defence mechanism hinges on the dynamic production of antibodies by B cells through V(D)J recombination, a process that assembles modular genomic segments to yield a repertoire of antibodies diverse enough to combat a wide array of threats presenting a diverse set of antigens. Antibodies are typically composed of two identical heavy (H) and two light (L) chains, forming a Y-shaped molecular structure. Antibody isotypes (classes) are defined by the identity of the H chain. While mammals have five heavy chain isotypes with different effector functions [1], teleosts, the largest group of bony fish possess only three (IgM, IgD and IgT). Among these, IgM is nearly universal to all jawed vertebrates, mainly present in the blood and extracellular space, but to a lesser extent also on mucosal surfaces [2]. Previous studies have confirmed the presence of IgD in the channel catfish [3] and later in other teleosts [4–8], suggesting that it is also an ancient isotype [9]. Bony fish have also evolved a unique isotype, initially discovered in zebrafish [10] and rainbow trout [11] and subsequently confirmed in many teleost species and holostei [12], with a predilection for mucosal defence in for example gut and skin [2, 13]. However, notable exceptions have also been found, including the gadidae (Atlantic cod) and siluriformers (catfish) lineages, which appear to lack the IgT isotype.

The genomic organisation of immunoglobulin heavy chain loci in teleosts generally rests on the same principles as for mammals. Commonalities include the translocon organization of the IgM locus, which features a sequence of multiple V, D, J, and C genes. IgD constant (C) genes are located at the immediate 3' end of the IgM C genes, and expressed by alternative splicing, although it involves the retention of the first constant-domain exon of IgM, resulting in a chimeric antibody molecule [3]. However, IgD C genes in different species have a considerable variation in the number of domains, ranging from 5 to 17 domains [4, 14, 15] preceding the transmembrane domain, resulting in the receptor structure VDJ-C_µ1_-(C_δ_)_n_-TM, while IgM chains usually have 4 C_µ_ domains. Frequent duplications and deletions of IgD exons suggest that this has undergone extensive rearrangements to meet the diverse functional requirements.. The teleost-specific isotype, IgT, is often located between the variable (V) and C genes of IgM in a configuration reminiscent of the mammalian T cell receptor A/D loci [10]. Most IgT genes have four Cτ exons irrespectively if they are membrane bound or secreted [16]. Notably, chimeric IgD/IgT and IgM/IgT receptors were found in European sea bass [17] and the common carp [18], respectively. There is a considerable variation in the number of Ig genes and loci in teleosts. For example, the Atlantic salmon exhibits a duplication of the IgH locus, retained from the whole genome duplication of the salmonids, a genetic strategy hypothesised to expand antibody repertoire diversity [19]. Triplicate heavy chain loci were found in the channel catfish [20], albeit for two loci, the IgM C genes are pseudogenes and only IgDs are functional. The genetic diversity among VH chains is vast; for example, the Atlantic salmon boasts 18 VH families with approximately 300 alleles, whereas the torafugu and the stickleback present merely 3 and 4 VH families, encompassing 34–49 V genes, respectively [21].

Mammalian light chains are either lambda or kappa isotypes, whereas fish display a more complex and diverse set of light chains and corresponding evolutionary history [22]. Four isotypes have been described in elasmobranchs, with several concurrent nomenclatures. Type I, also called sigma-2, was originally discovered in cartilaginous fish. Type II shows signs of homology to lambda chains and type III, or L1, to kappa chains. A sigma homologue, reminiscent of that found in *Xenopus*, has also been detected in fish (Type IV or L2). Recent analysis has further classified lambda-like chains (Type II) into distinct isotypes, lambda and lambda-2 [23]. An alternative classification is presented in the IMGT database, denoting teleost light chains as iota and classifying them into four subgroups namely IGIC1 to IGIC4 [24], corresponding to L1, L2 and L3 and lambda. Notably IGIC4 has only been found in the Atlantic cod to date. Contrary to the heavy chains, the genomic organization of light chains in fish involves numerous mini-clusters with a (V-J-C)_n_ configuration in tandem within the genome [25–27]. These clusters exhibit varying orientations of genes, with inverse orientation predominantly observed in kappa light chains, which facilitates inversional rearrangement instead of deletion, maintaining a larger set of germline diversity for secondary rearrangements. The number of IgL mini-clusters also varies significantly among fish species and isotypes, with kappa light chains generally being the most abundant, leading to a varied level of germline diversity.

The Atlantic cod presents an intriguing paradigm in the context of the adaptive immune system. Historical data on its weak specific antibody response have been linked to a suspected deficiency in MHCII [28], a hypothesis that was corroborated upon the publication of its complete genome in 2011 [29]. The genome analysis revealed not only the absence of MHCII but also the lack of the invariant chain and a truncated, non-functional CD4, implying a compromised T cell-dependent antibody response pathway. Additionally, the AID protein in Atlantic cod was shown to be inactive [30]. The complete lack of the MHCII-CD4 pathway as well as the absence of AID activity preclude the occurrence of somatic hypermutation and affinity maturation in Atlantic cod. Finally, the Atlantic cod lacks the main teleost mucosal isotype IgT locus in its entirety. Prior to the publication of the complete genome, the Ig loci were probed and sequenced from cDNA and genomic libraries in search for mechanistic explanations for the weak antibody response in Atlantic cod. In these works, four heavy chain V families, with an estimated 50 VH genes were established based on sequence similarity [31, 32] in translocon organization, with the C_µ_ and C_δ_ genes, the latter of which has shown to have a peculiar structure with deleted δ_3-6_ exons and duplicated δ_1-2_ domains [5]. All VH families were expressed in unchallenged fish [32], despite large differences in number of genes per family. Mini-clusters of light chains have also been described for κ and λ-like chains [26, 33], the former showing inverted VL orientation compared to JL and CL. Germline diversity was shown to be comparable to other species for both heavy [32] and light [34] chains. However, repertoire analysis was constrained by the absence of complete genomic data and advanced sequencing technologies. Description of the complete immunoglobulin loci and an exhaustive list of germline repertoire remained elusive so far. The aim of this study is a complete annotation of the germline IgH and IgL loci of the Atlantic cod and extends to characterizing the diversity of combined IgM/IgD repertoire as well as all light chain isotypes at the CDR3 level in unchallenged fish. The objective is to unravel the translation of germline diversity into functional antibody repertoire diversity, shedding light on the consequences of the Atlantic cod’s unique adaptive immune configuration.

## Methods

### Identification of BCR genes in gadmor3.0

Regions of interest were identified in the latest reference of the Atlantic cod genome, gadmor3.0, with a sequence similarity search of known V- and C-genes from IMGT [24] using tBLASTn, with lenient e-value cutoffs (0.1). Genomic regions returning hits for both V and C genes were analysed further. A more specific similarity search with tBLASTn with a 0.00001 cutoff was conducted for V and C genes, along with the addition of J genes from IMGT, employing a 0.1 e-value cutoff due to their shorter sequence length. This defined the potential genes in the regions of interest. Additional data, including whole transcriptome data from a prior study [35], assisted in delineating intron–exon boundaries as there is a several-fold drop in the number of mapped reads for the unspliced RNA compared to the spliced variant. A 10 million read subsample was mapped to the regions of interest using STAR [36] with default parameters in a splice-aware manner, after trimming with Trimmomatic0.36 [37], with a minimum quality score of 20 over a sliding window length of 4. Splice sites underwent manual validation for canonical splice signals (GT/AG). Recombination signal sequences (RSS) were annotated through canonical heptamer and nonamer sequence (CACAGTG and ACAAAAACC respectively) searches. Comparing 28 or 39 nucleotides at the end of V genes lacking canonical RSS sequences to V genes yielded additional genome-specific RSS signals. D genes were annotated by locating two sets of proximal RSS signals situated between V- and J-genes. Data from all sources were visualized in IGV [38] and manually integrated for the final annotation. Translocons containing heavy chain genes were delineated by the 5’ most V gene and the end of the corresponding downstream C genes, and denoted translocon 1, 2 and 3. Gene nomenclature was defined in accordance with the IMGT naming schemes. However, in case of heavy chain V genes, the translocon number was added to the gene name between the first number that denotes the V-gene family and the last number that is unique for each V-gene. In case of light chains, the nomenclature was defined so that V, J and C genes from the same mini-cluster always have the same number. In case of a deletion of a J or C gene, the corresponding number is skipped. Duplicated genes received an additional suffix. Gene sequences were extracted, aligned with the MUSCLE algorithm [39] and used in IMGT V-quest [40] and Collier de Perles [41] for the identification of CDR and framework regions. Phylogeny for V- and C-genes was created using MEGA11 [42] with the neighbour-joining method [43] and 1,000 bootstrap replicates, using a diverse set of reference sequences [23]. The P-distance method quantified evolutionary distances, and ambiguous sites were removed in a pairwise fashion.

### 5’RACE sequencing of immunoglobulin repertoires

Atlantic cod were purchased from NOFIMA (Tromsø, Norway) and reared at the NIVA facility (Solbergstrand, Norway) as described earlier [44]. Spleen was harvested from 6–12-month-old cod (weight 50–500 g), transported in RPMI media on ice and single-cell suspension was prepared and frozen within 6 h of organ harvest. Two to ten million single spleen cells from each fish were resuspended and homogenized in RLT plus lysis buffer (Qiagen) and kept frozen at –70 °C until RNA isolation. Total RNA was purified from the spleen cells using the RNeasy Plus Mini Kit (Qiagen) according to the manufacturer’s instructions. cDNA libraries of immunoglobulin heavy and light chains were prepared by 5’ RACE-PCR using template switch and nested C-gene specific primers located close to the V(D)J-junction. First strand cDNA was made from approximately 500 ng total RNA in the presence of a template-switch oligo (TSO) that enabled the incorporation of a universal priming site at the 3’-end of the first-strand cDNA corresponding to the 5’-end of mRNA. The first-strand synthesis reaction consisted of 1 µM oligo-dT primer (all primer sequences are found in Supplementary Table 1), 1 mM dNTP, 1 × RT buffer, 2.5 mM DTT, 8 mM MgCl_2_, 1 µM betaine (Merck), 0.8 U/µL Murine RNase Inhibitor (NEB), 1 µM TSO, and 8 U/µL Superscript II (Invitrogen) in a total volume of 40 µL. The reaction mix was incubated for 90 min at 42 °C, followed by an inactivation step of 72 °C for 15 min. In the first of total three semi-nested PCR reactions, 1.5 µL first strand cDNA was used as template where both Ig heavy and Ig light chains were targeted in the same PCR reaction. The first PCR reaction contained also 40 nM of STRTfwd_long primer, 200 nM each of the primers STRTfwd_short, IGHC_O, IGLC_O1, IGLC_O2, IGLC_O3, 200 µM dNTP, 1 × Phusion high-fidelity buffer, 0.1 µL of Phusion polymerase (ThermoFisher) in a total reaction volume of 15 µL, and run with the cycling condition of denaturation 1 min at 98C; five cycles of (10 s at 98 °C, 60 s at 72 °C); five cycles of (10 s at 98 °C, 30 s at 70 °C, 40 s at 72 °C); eight cycles of (10 s at 98 °C, 30 s at 68 °C, 40 s at 72 °C); and final extension of 4 min at 72 °C. In the following second PCR, the Ig heavy and light chains were amplified in separate reactions each with 1 µL product from the first PCR reaction as template. In a total volume of 10 µL, the second PCR reactions contained 40 nM of STRTfwd_long primer, 200 nM each of the primers STRTfwd_short and IGHC_M (for Ig heavy chain second PCR); or IGLC_M1, IGLC_M2, IGLC_M3 primers (for Ig light chain second PCR); 1 × KAPA HiFi HotStart ReadyMix (Roche), and run with the cycling condition of denaturation of 2 min at 95 °C; 10 cycles of (15 s at 98 °C, 30 s at 65 °C, 40 s at 72 °C); and final extension of 5 min at 72 °C. In the third PCR, 1 µL product from the second PCR reaction was used as template in each of the four third PCR reactions, one for Ig heavy chain and three reactions for the three Ig light chain subclasses. In a total volume of 15 µL, each third PCR reaction contained 200 nM each of the primers R2_STRT_bulk and IGHC_I (for Ig heavy chain); or IGLC_I1, IGLC_I2, IGLC_I3 primers (for Ig light chain) and run with the same KAPA polymerase and cycling condition as the second PCR reactions. Each of the primers used in the third PCR contains a unique 6-nt barcode that identifies the sample origin, as well as portions of oligo sequences that are necessary for Illumina sequencing platforms. In the final library PCR reactions, 1.5 µL product from the third PCR reaction was used as template in total 20 µL reaction containing 200 nM each of the R1_library and R2_library primers containing sequences that completed Illumina sequencing adapters, 1 × KAPA HiFi HotStart ReadyMix and run with the cycling condition of denaturation 2 min at 95 °C; 10 cycles of (15 s at 98 °C, 30 s at 60 °C, 40 s at 72 °C); and final extension of 5 min at 72 °C.

The final library product from all samples were pooled separately for each chain subclass and purified with 0.65 × volume of AMPure XP beads (Beckman Coulter) according to manufacturer’s instructions. The purified products were eluted from the beads in 17 µL water, added with gel loading buffer and loaded directly onto a 1.2% agarose gel. Visible bands between 600–700 bp were excised out of the gel and purified using the Monarch DNA gel extraction kit (NEB). The gel-purified products were quality checked on BioAnalyzer (Agilent) and then submitted to 150 bp pair-end sequencing on ½ lane of an Illumina NovaSeq SP chip. Read 1, which has better overall quality, was used to sequence the CDR3 in the 3’-5’ direction starting from the first 24–42 bp of the 5’-end of the C-genes. Read 2 sequences the 5’-end of the V-genes starting from the 5’-UTR. The sequencing was performed at the Norwegian Sequencing Centre at the Oslo University Hospital.

### Analysis of BCRH and BCRL repertoires

The MiXCR [45] pipeline was used for sequence mapping, assembly, and quantification, with the *analyze generic amplicon* preset and default parameters, but only functional chains were used in downstream analyses (parameter –only-productive). Additionally, since C genes can not be identified due to sequence similarity, –dont-split-clones-by C parameter was used. Finally, since the whole receptor sequence could not be reconstructed due to short read lengths, –assemble-clonotypes-by CDR3 was employed to collapse receptors based on their CDR3 nucleotide sequence. The reference database for MiXCR was formatted with RepSeqIO. Coordinates were manually incorporated due to automatic identification failures stemming from large evolutionary distances between cod and the mammalian reference database. The immunarch [46] package in RStudio (RStudio Team, [47]) (R version 4.2.2, [48]) was employed for the analysis of MiXCR outputs. Receptors with less than 5 assigned reads were discarded. Lastly, ggplot2 (3.4.4, [49]) facilitated visualization.

## Results

### Identification of IgH and IgL genes in gadmor3.0

**Fig. 1:**
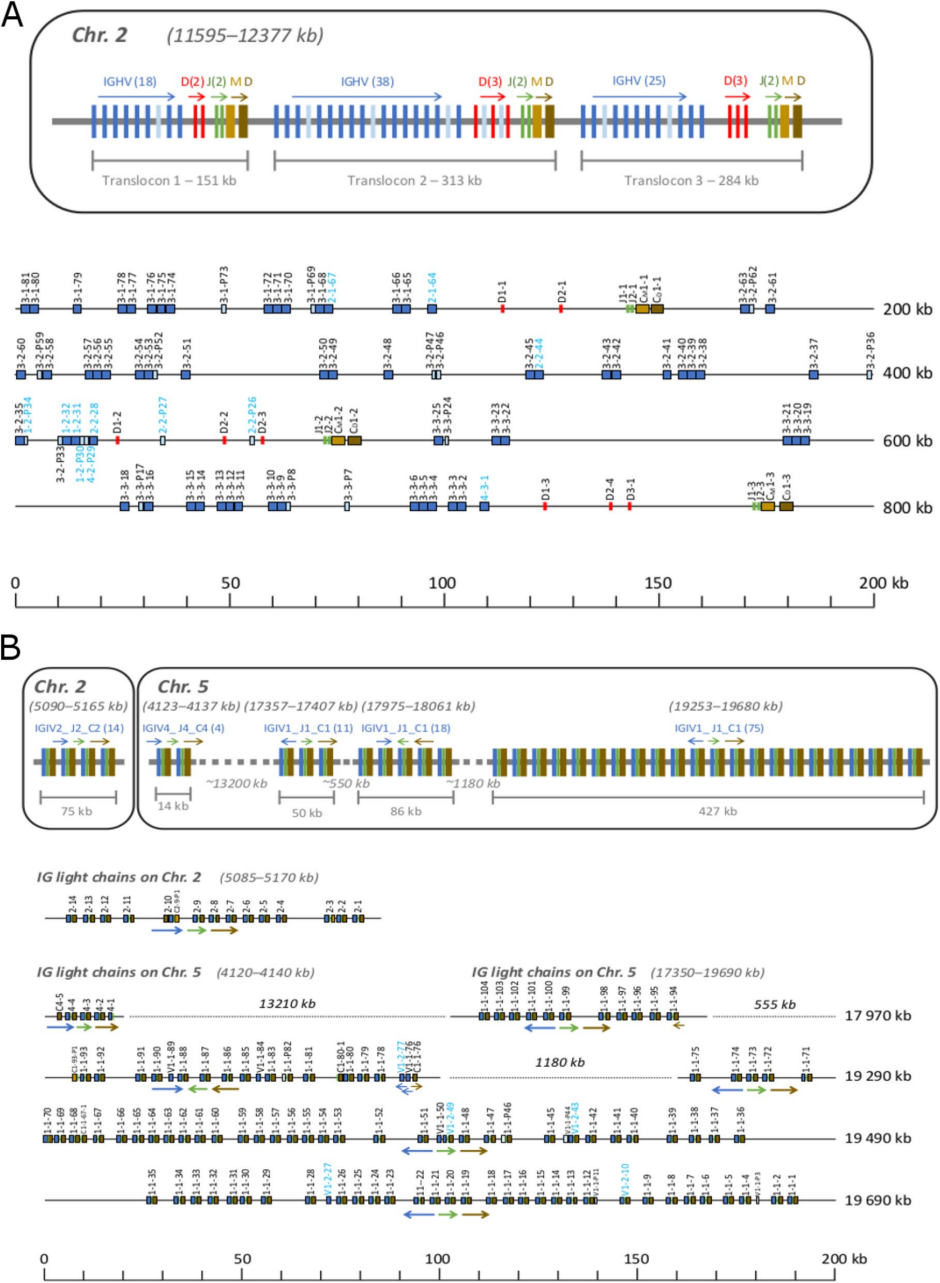
Schematic representation of IGH and IGL loci in the genome of the Atlantic cod. **A** Schematic (top) and detailed (bottom) map of the IGH locus on chromosome 2 (11,595–12,377 kb). In the schematic the distances are not to scale and the number of blue bars is about one half of the number of V-genes. Numbers in the brackets indicate the total number of genes including pseudogenes. All genes are in the forward orientation indicated by the arrows. The V-genes are in blue of which the pseudogenes are in a lighter shade. D-genes are in red, and J-genes are green. IGHM genes are light brown and IGHD genes are dark brown. In the detailed map the numbers refer to V-gene names unless otherwise specified. Names of V-genes in families other than the largest family 3 are depicted in light blue for easier identification. **B** Schematic (top) and detailed (bottom) map of the IGL loci on chromosome 2 (5,085–5,170 kb) and chromosome 5 (4,120–4,140 kb and 17,350–19,690 kb). In the schematic the distances are not to scale. Numbers in the brackets indicate the total number of complete sets of V-J-C mini-clusters. In the detailed map the V-genes are in blue of which the pseudogenes are in a lighter shade of blue. J-genes are green, C genes are dark brown, and pseudogenes are light brown. The numbers refer to V-J-C mini-clusters unless otherwise specified. Names of V-genes in family V1-2 are depicted in light blue for easier identification. Thick arrows indicate the prevalent orientation of the V-genes (blue), J-genes (green) and C-genes (brown) in the locus, whereas thin arrows indicate genes that have diverging orientation from the main rule

Located on chromosome 2 between bases 11,595,000 and 12,380,000, the complete set of immunoglobulin heavy chain (IgH) genes is organized into three sequential translocons of comparable size (Fig. 1A). Each translocon encompasses V, D, J, and C genes, all in the same orientation. The functionality of these genes is ascertained based on three criteria: V genes require the presence of recombination signal sequences (RSS), a promoter, and an absence of frameshifts or stop codons, while J and C genes must be devoid of frameshifts and stop codons. Both D and J genes had intact RSS sequences to be considered functional. The first translocon contains 18 V genes (two being pseudogenes) and 2 D genes. The second is composed of 38 V genes (of which 12 pseudogenes) and 3 D genes, and the third 25 V genes (4 pseudogenes) alongside 3 D genes. Each translocon contains 2 J genes and 2 C genes. The C_δ_ gene is found immediately downstream of C_µ_ as previously described, both chains sharing the first exon of IgM (C_µ1_) and alternative splicing determines the expressed isotype [5]. Detailed information about immunoglobulin heavy chains is presented in Supplementary Table 2. Several V genes showed 100% sequence similarity to already described V genes in the IMGT database. Identifiers of these V genes were also included in Supplementary Table 2.

Diverging from the organization of IgH genes, immunoglobulin light chain (IgL) genes are located on chromosomes 2 and 5, displaying a distinctive mini-cluster configuration (Fig. 1B, Supplementary Table 3). This structure consists of a set of sequentially repeated units containing single V, J, and C genes. Chromosome 2 (between bases 5,087,000 and 5,170,000 hosts 14 V, 13 J, and 15 C genes. All genes are functional, except one C gene (IGIC2-13*01) due to an internal stop codon. On chromosome 5, in a mere 15 kbases starting at 4,120,000, a compact arrangement reveals a total of 12 genes, distributed equally among V, J, and C genes. The most gene-rich region is on chromosome 5 (between bases 17,354,000 and 19,680,000), where 104 V genes (of which 6 pseudogenes), 98 J and 99 functional C genes reside. Consistent with previous findings [26], most V genes on chromosome 5 were found in the opposite orientation of the J and C genes. Intriguingly, a subset of five V genes align in the same forward direction as J and C genes, using recombination without retaining the inverted segment. Distances between mini-clusters vary based on their chromosomal location. Clusters on chromosome 2 have on average the shortest, 1.5 kb, distance between mini-clusters (375–3,298 bp). The average distance between the first set of mini-clusters starting at 4.12 Mb on chromosome 5 is 6.5 kb (5,320–8,890), and the second set of mini-clusters at 17.35 Mb is 3.7 kb (712–9,573 bp). The difference in VL gene orientation to JL and JC genes and inter-cluster distances suggest that the frequency of inter-cluster recombination may differ between isotypes, affected by the germline configuration. Further similarity searches did not reveal any other genomic loci with immunoglobulin genes, suggesting the complete catalogue of Ig genes have been identified in the Atlantic cod genome. The total number of identified immunoglobulin genes are presented in Table 1.

**Table 1:** Overview of the number of genes within each subtype. The number of pseudogenes are shown in brackets

	**V**	IGHV1	IGHV2	IGHV3	IGHV4	**D**	**J**	**C**
**IgH total**	**81 (18)**	4 (2)	6 (2)	9 (13)	2 (1)	**8**	**6**	**6**
Translocon 1	18 (2)	0	2	16 (2)	0	2	2	2
Translocon 2	38 (12)	4 (2)	4 (2)	29 (7)	1 (1)	3	2	2
Translocon 3	25 (4)	0	0	24 (4)	1	3	2	2
**IgL total**	**122 (6)**	-	-	-	-	**-**	**115**	**118 (1)**
IgL Kappa	104 (6)	-	-	-	-	-	98	99
IgL Sigma	14	-	-	-	-	-	13	15 (1)
IgL Lambda-2	4	-	-	-	-	-	4	4

### Classification of Atlantic cod immunoglobulin germline sequences

Nomenclature of both immunoglobulin heavy and light chains were established in accordance with the IMGT format, based on previously discovered Atlantic cod sequences. V gene family names also adhere to previously established classification [5, 31, 34, 50]. However, gene identifiers were assigned according to chromosomal location instead of order of discovery, as was for previously described genes. Each gene was assigned a unique identifier, along with the translocon it originates from, since they represent separate entities and future analyses may uncover different allelic variants. IgH isotypes were clearly established using sequence similarity analysis, confirming the presence of IgM and IgD, but not IgT. Phylogenetic analysis of translated coding regions of IgH V genes showed four distinct V gene families (Supplementary Fig. 1), with less than 80% sequence similarity between families that is consistent with previous classification. The most prominent family is IGHV3 with 69 of 81 members, whereas other families contain a mere 2-6 members each. V genes from the IGHV1 family are all located in the second translocon of IgH genes, while V genes from IGHV2 are at the 3’ end of both translocon 1 and 2, close to IGHD genes. IGHV4 has only two members, a functional V gene right before the IGHD genes in the third translocon and a non-functional V gene close to the 3’ end of V genes in the second translocon.

Previous exploration of the immunoglobulin light chains in fish uncovered a complex phylogeny [22] with five [23] ancestral isotypes. A kappa-like orthologue group sometimes further separated into L1 and L3 in some, but not all teleost fishes, even though their phylogeny is intertwined [33, 51]. L2, also referred to as sigma, with no homologue in mammals, and a lambda-like group, which was recently subdivided into lambda and lambda-2, as the teleostean lambda sequences appeared to be paralogs to lambda and kappa [23]. The last isotype is sigma-2, an isotype initially considered specific to cartilaginous fish, but later identified in holosteans, polypterids and turtles as well. Classification of light chain isotypes mainly relies on sequence homology, even though in some instances taxonomic relationships are stronger than isotypes [52]. C genes were assigned isotypes based on maximum likelihood phylogenetics based on previous analyses [33] using reference sequences from other fish species from GeneBank (Supplementary Fig. 2). The analysis revealed genes on chromosome 2 belonging to the L2 (sigma or Ig-iota-2) isotype, genes on chromosome 5 to the lambda-2 (Ig-iota-4) isotype, and on a different part of the chromosome to the L1 (kappa or Ig-iota-1) isotype. A separate phylogeny of the V genes' coding regions delineated their family structure (Supplementary Fig. 3), revealing that V gene families align with isotypes. However, the kappa V genes bifurcate into two distinct families, IGIV1-1 and IGIV1-2. Interestingly, the IGIV1-2 family, with its five members, maintains the same orientation as their corresponding J and C genes, deviating from the majority of IGIV1-1 V genes. We have used gene nomenclature that follows the IMGT classification, where the isotypes are denoted Ig-iota (IGI). All the parallel naming schemes and light chain classifications are presented in Table 2 for clarity.

**Table 2:** Correspondance of light chain classification used in this and other publications

IMGT chain type	IMGT subgroup	Chain type I	Chain type II	Chain type III
IG-Light-Iota-1	IGIC1	L1(L3)	kappa	Type III
IG-Light-Iota-2	IGIC2	L2	sigma	Type IV
IG-Light-Iota-4	IGIC4	–-	lambda	Type II

### Genomic features: RSS and promoters in immunoglobulins

A comprehensive sequence analysis of the IgL genes highlights a strong conservation in their promoter structures. Regardless of the expansive proliferation in kappa chains, elements like the TATA-box and the octamer manifest substantial conservation across isotypes. Octamer and TATA-box sequences and the number of nucleotides between them are the same for almost all kappa V genes. The same can be observed for the other isotypes, with their own characteristic octamer and TATA-box sequences (Supplementary Table 3).

Since RSS orientation is conserved among isotypes, it is also a frequently used method for supporting classification based on sequence homology. Kappa, sigma and sigma-2 IgLs have V12-23J structure for their immunoglobulins, while lambda has V23-12J. Indeed, cod lambda-2 (Ig-iota-4) light chains feature a 23 nt spacer in their V gene's RSS and a 12 nt spacer in the J gene's RSS, standing in contrast to the reversed configuration in kappa (Ig-iota-1) and sigma (Ig-iota-2) chains, supporting their classification. Notably, only a single IgL V gene has a non-canonical RSS sequence. As seen for promoter sequences, RSS also exhibit pronounced conservation based on the isotype. The canonical CACAGTG heptamer and ACAAAAAC(C/A) nonamer prevail in most V genes, flanking a highly conserved spacer sequence. The heavy chains on the other hand, display a more varied landscape in terms of promoter and RSS structures. Out of all 81 V genes, merely 38 and 15 adhere to the canonical RSS heptamer sequences of CACAGCA and CACAGTG, respectively, while others diverge to non-canonical heptamers. The nonamers in IgH predominantly retain the canonical ACAAAAACC sequence as well. In stark contrast to the varied RSS in IgH V genes, both D and J genes share conserved RSS structures. Notably, some V genes (IGHV3-1–72*01, IGHV3-1–71*01, IGHV3-1–70*01 and IGHV3-3–5*01, IGHV3-3–4*01 respectively) are clustered within a mere 1.5 kb from each other, sharing a TATA-box and an octamer.

### Germline variability of immunoglobulin genes

The absence of MHCII and CD4 and the inactivation of the AID gene [30] in Atlantic cod provides strong evidence against the presence of somatic hypermutation in this species. Consequently, it relies exclusively on deletions, and P- and N nucleotide additions and the combinatorial variability of its VDJ genes to diversify its immunoglobulin sequences. Hence, the immunoglobulin sequence variability is intrinsically tied to the germline sequence diversity. Given the disproportionate size of IGHV3 and IGIV1-1 families, they contribute most of the germline-encoded V-gene sequence diversityto their respective repertoires. Translation of the nucleotide sequence highlights a comparable sequence diversity between the largest families of light and heavy chain variable genes, with light chains showcasing a more concentrated variability in the complementarity-determining region (CDR, Supplementary Fig. 4A and 4B). An interesting observation is made in the heavy chain variable region CDR3s, where six V sequences harbour in-frame stop codons immediately prior to the recombination site (IGHV3-76*01, IGHV3-71*01, IGHV3-54*01, IGHV3-10*01, IGHV3-5*01, IGHV3-2*01), yet can still produce functional chains given a deletion of at least two nucleotides.

Further probing into the germline sequences unveiled that all IGHD genes share a characteristic stretch of guanines, with the majority of their sequence being conserved. Their contribution to diversity predominantly stems from variations in chain lengths (Supplementary Table 3). Conversely, the six heavy chain J genes can be categorized into two families (IGHJ1 and IGHJ2), each exhibiting no sequence variability within the same family. This renders differentiation between individual genes within each J family unfeasible. Coupled with the strong conservation observed in C genes, the prospect of tracing inter-translocon recombination through sequencing the antibody transcript repertoire remains elusive. Although there are 98 immunoglobulin light chain J genes, they contribute minimally to sequence diversity (Supplementary Fig. 4C). Overall germline J gene sequence variability does not seem to contribute significantly to the diversification of the immunoglobulin sequences, be it for heavy or light chains.

### Characterization of immunoglobulin repertoire by 5’RACE sequencing

To comprehensively assess the immunoglobulin heavy and light chain repertoire in Atlantic cod, total RNA was isolated from the spleen of six untreated fish and subjected to 5’RACE sequencing of the IgH and IgL cDNA using specific primers for all isotypes of light chains and Ig_µ1_ common for both heavy chain isotypes. We included an additional technical replicate (F9020a and F9020b) to ensure the veracity of the results. Sequencing was conducted with the Illumina HiSeq platform for four samples from each fish to cover the different isotypes. Primers and sequencing statistics are presented in Supplementary Tables 1 and 4 respectively.

**Fig. 2:**
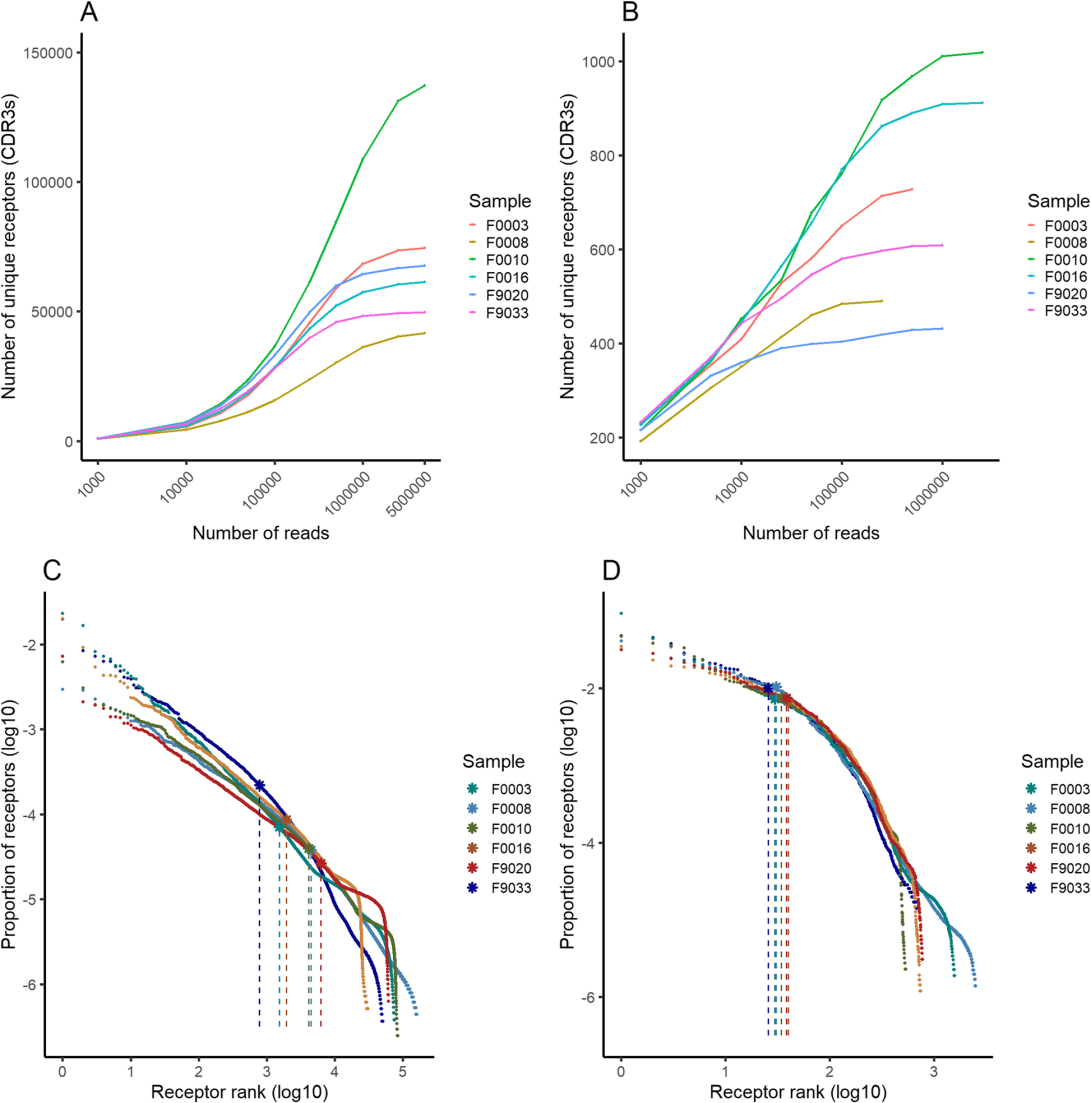
Number of unique receptors based on sequencing depth and abundance-based receptor rank and frequency for IgH and IgL iota-1 (kappa) chains. Number of unique receptors identified at the CDR3 amino-acid sequence level in relation to mapped sequencing reads for IgH (**A**) and IgL kappa (**B**) receptors. Subsamples of reads were created from mapped reads for each sample. Sampling depth was adjusted for each sample according to total number of mapped reads. Abundance-based receptor rank and proportion of repertoire of IgH (**C**) and iota-1 (kappa) light chains (**D**). Each point represents an individual receptor, stars denote D50 values for each sample. All axes are logarithmic. D50 values and corresponding vertical lines were jittered slightly for better visibility

Following assembly of unique, functional heavy and light chain sequences at the CDR3 nucleotide level, a diversity ranging from 40,000 to 144,000 unique heavy chain CDR3s was identified in each individual fish (Supplementary Table 4), establishing a conservative lower bound estimate of diversity. A comparative analysis revealed that light chains exhibited two orders of magnitude lower diversity compared to heavy chains, despite greater genomic loci count for IgL kappa (Fig. 2A and B). Sigma chains had lower diversity with around 100 unique CDR3s. Strikingly, the diversity of lambda-2 chains was below 10 across all fish (Supplementary Fig. 5A and B). Subsampling for each chain type revealed a saturation in the number of unique CDR3s discovered at the sampling depths used in our experiments. This suggest that the employed sequencing depth was sufficient to capture most light chains and a good representation of heavy chains, especially for higher abundance receptors.

**Fig. 3:**
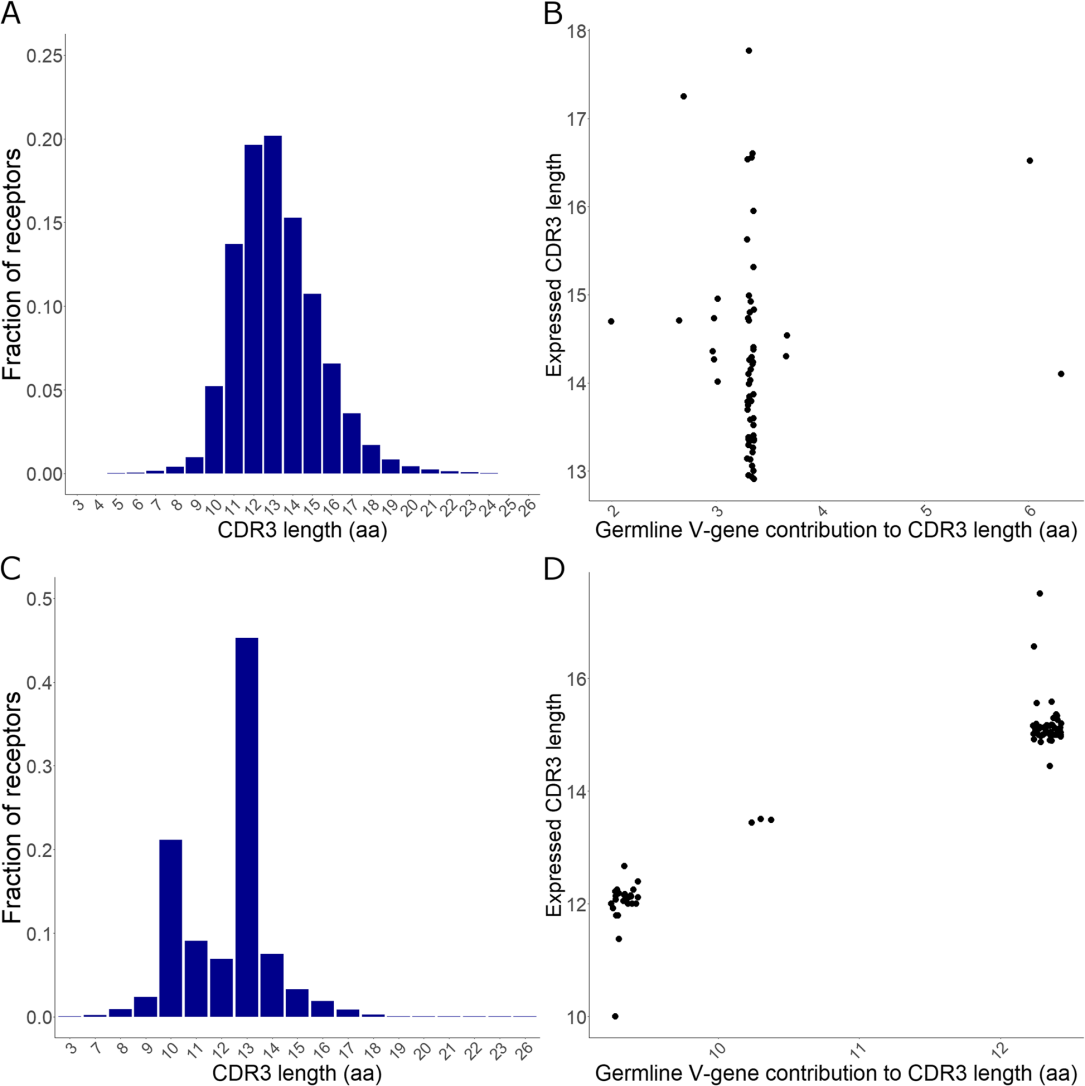
CDR3 length distribution of immunoglobulin heavy (**A**) and iota-1 (kappa) light chains (**C**) and relationship between V-gene germline and expressed CDR3 lengths (**B** and **D**, respectively). All samples were combined for calculating CDR3 length distributions. Germline V-gene CDR3 length was calculated by taking the number of CDR3 nucleotides from germline sequences and dividing them by 3. No significant correlation was found between IgH germline V-gene and expressed CDR3 lengths (Pearson’s R = 0.06, P = 0.63), while very strong correlation was observed in case of kappa chains (Pearson’s R = 0.96, P < 2.2*10^–16^). Small jitter was added to plots **B** and **D** for better visibility

Further characterization of the repertoire encompassed the establishment of baseline D50 values, revealing that for heavy chains (Fig. 2C), kappa (Fig. 2D), and sigma light chains (Supplementary Fig. 5C), 2–10% of CDR3s represented 50% of the repertoire, regardless of total diversity of the isotype, while for lambda-2 chains, 1–2 receptors encompassed 50% of the repertoire (Supplementary Fig. 5D). Analysis of CDR3 length highlighted an average of 13.26 amino acids for heavy chains (Fig. 3A), which is larger than previously observed [32]. The germline V-gene CDR3 length does not significantly impact the VDJ-recombined CDR3 length where varying V-gene germline CDR3 lengths converge to yield similar receptor CDR3 lengths (Fig. 3B), predominantly influenced by nucleotide deletions. No significant correlation was found between CDR3 length and V gene usage (Pearson’s R = -0.04, P-value = 0.74). In contrast, kappa light chains exhibited an average CDR3 length of 13.11 across fish, with two distinct peaks (Fig. 3C) and a strong correlation to germline V-gene CDR3 lengths (Fig. 3D), and markedly less variation from insertions and deletions at junctions compared to heavy chains. Sigma isotypes show little variation in CDR3 lengths (Supplementary Fig. 6A) with complete uniformity of IGLV germline CDR3 length. In contrast, although lambda-2 V genes all have the same IGLV germline CDR3 lengths, two distinct peaks were observed in the CDR3 (Supplementary Fig. 6B) lengths of the expressed receptors. This is exclusively the result of using different J genes.

**Fig. 4:**
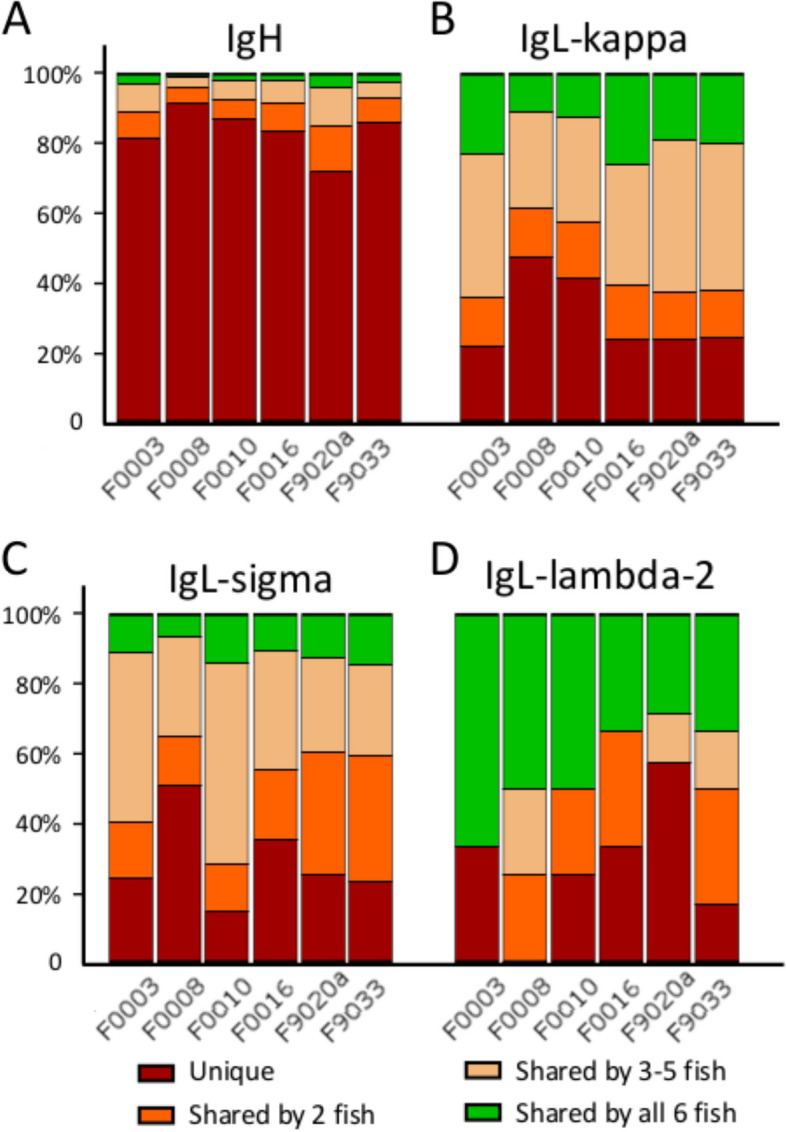
Proportion of the repertoire unique to each fish and the proportion of the repertoire shared between two, widely shared between several fish and shared by all per immunoglobulin isotype. Colours denote the proportion of the levels of sharedness of the repertoire

Next, sizes of the unique repertoires and shared between fish were estimated, with only one of the technical replicates used in this analysis (F9020a). Fish used in the experiment are either siblings or half-siblings reared in an identical environment, thus some degree of overlap between their BCR repertoires is expected. A large percentage of the repertoire was indeed widely shared across all immunoglobulin heavy chains, ranging from 2–7% of the repertoire of individual fish, a total of 1,073 shared heavy chain CDR3s of the 353,602 CDR3s detected in all fish. Additionally, on average 19% of the heavy chain repertoire was shared between at least two individuals. Conversely, between 70 to 87% of the repertoire was unique to individual fish (Fig. 4A). Notably, we observed 34% shared heavy chain repertoire between technical replicates in which two independent RT-PCR samples were generated from the same RNA sample. Immunoglobulin light chains showed an even higher degree of repertoire similarity (Figs. 4B-D), attributed to their lower level of total diversity. 111 kappa light chains are shared between all fish, 10% to 25% of the total repertoire and only an average of 29% of the repertoire was unique to any individual. Despite an order of magnitude lower total diversity, IgL sigma chains show a comparative level of similarity: only 14 chains are common among all fish, which is between 6 and 14% depending on the size of the repertoire. Additionally, an average of 28% of the repertoire is unique to each fish. Even though only two lambda-2 light chains are widely shared among all individuals, this constitutes 25% to 67% of the overall repertoire due to the extremely limited diversity. Notably F0008 has no unique immunoglobulins even among the six fish investigated. Technical replicates showed 66% similarity for kappa chains, 73% similarity for sigma chains and 78% similarity for lambda-2 chains respectively, with notably 100% of lambda-2 receptors of sample F9020b were found in sample F9020a.

**Fig. 5:**
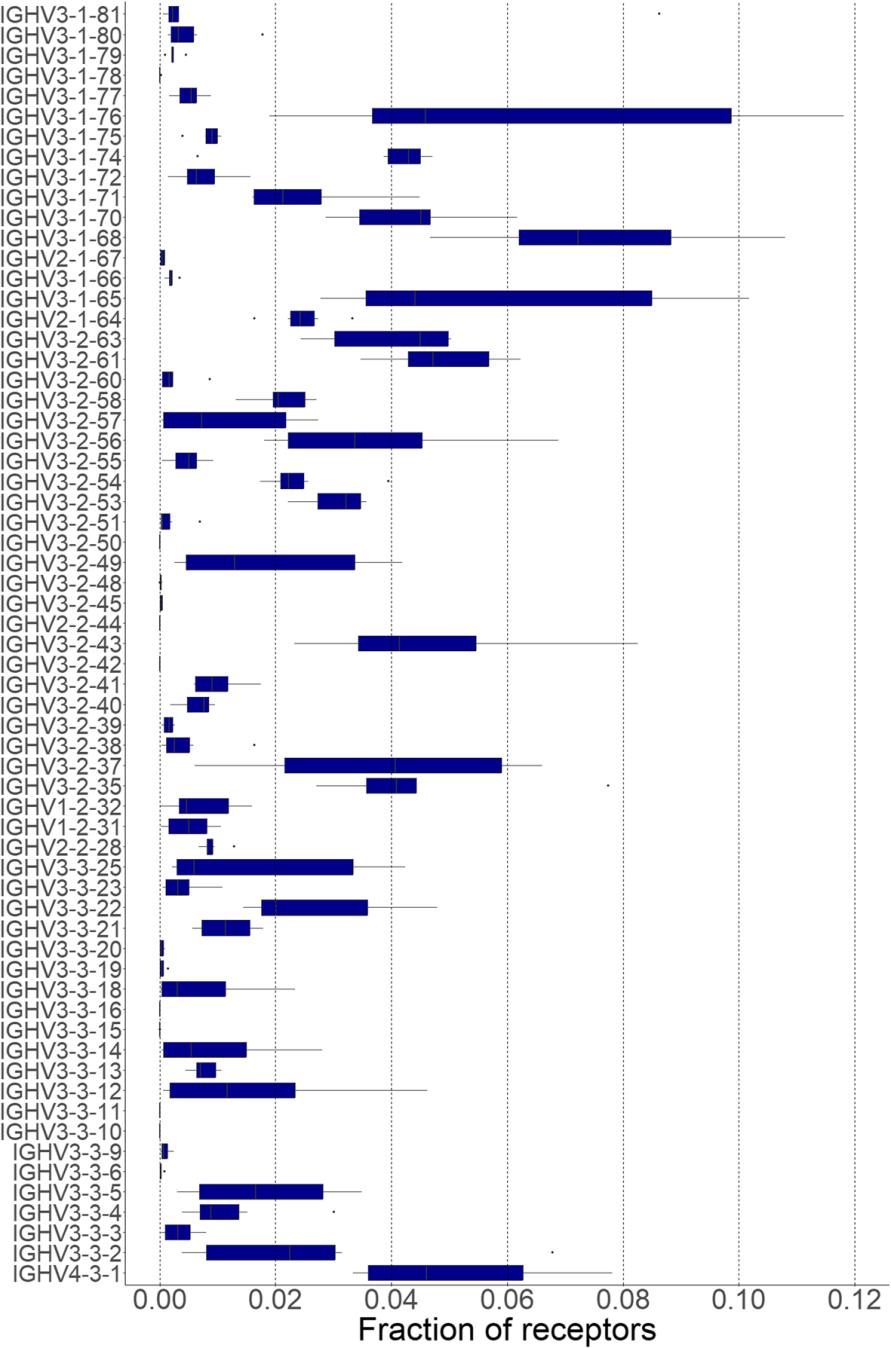
V gene usage in immunoglobulin heavy chains. Genes are ordered based on family and gene identifiers. All samples were used in calculation of gene usage, error bars represent standard deviation between individual fish

V-gene usage analysis showed consistent expression of all heavy chain V gene families, despite the small size of IGHV1, IGHV2 and IGHV4 families (Fig. 5). Similarly, most V genes were expressed to some extent, with an average correlation coefficient of 0.663 in V-gene usage across all fish. JH1 genes are predominantly used for heavy chains, with an overall JH1/JH2 ratio of 7.6. VH2 family shows an even higher average JH1/JH2 of 10.8. Our data shows more consistently higher JH1 gene usage than previously reported for the three small VH families [32]. Kappa light chains had both V gene families expressed, albeit with more genes exhibiting low expression (Supplementary Fig. 7), resulting in a 0.516 average Pearson correlation in V-gene usage, significantly lower than for heavy chains. Similarly, sigma chains had an average correlation of 0.573 in V-gene usage, with all genes expressed (Supplementary Fig. 8A).

Interestingly, preferential gene usage can be detected for lambda-2 chains. IGIV4-3 was predominantly used, while IGIV4-4 was expressed in only one sample (Supplementary Fig. 8B). Additionally, for J genes, a preferential usage of IGIJ4-2, the single longest J gene by six nucleotides for lambda chains, was observed. This gene was utilized in 12 out of 18 chains, while the other three sequence-wise identical J genes were employed in the remaining six receptors.

## Discussion

The Atlantic cod offers an interesting case study for examining the evolution of immunoglobulins in fish, particularly in the context of its distinct immune characteristics. It is a prolific species in the Atlantic, despite its inconsistent specific antibody response to antigens in experimental settings [28]. The Atlantic cod lacks MHCII, invariant chain, and has a truncated CD4 [29], in conjunction with loss of function of the AID protein [30] that mediates somatic hypermutation. This genomic architecture is expected to yield a constrained antibody repertoire diversity. Loss of diversity can be compensated by extensive gene and locus duplication coupled with sequence divergence to increase germline variability. Alternatively, the number of genes can also be reduced if the specific antibody response became less important in favour of other defence mechanisms. To test these potential scenarios, we aimed to annotate the full range of immunoglobulin loci in the Atlantic cod and to characterize the expressed repertoire across all isotypes in unchallenged fish. Previous studies provided partial insights, however, a comprehensive analysis integrating both aspects was lacking. Our groundwork presented here is a necessary fundament for immunoglobulin repertoire analyses using modern high-throughput approaches in Atlantic cod.

Whole locus duplications for immunoglobulin heavy chain loci are ubiquitous in teleosts. The Atlantic cod has a triplicate locus, forming three consecutive translocons on chromosome 2. In our study, 81 IGHV genes were annotated, encompassing 18 pseudogenes (22% of all chains), and categorized into four V-gene families based on sequence similarity. This number of V-gene families is relatively low compared to the total gene count, and the majority of V genes belong to the IGHV3 family. Nevertheless, the Atlantic cod has an intermediate position in terms of the number of heavy chain variable genes—fewer than salmon [19] or the rainbow trout, but more than torafugu or stickleback [21], and a moderate germline V gene diversity. It also possesses two distinct but highly conserved sets of J genes, for which the specific functions remain to be elucidated. Significantly, based on the complete locus description we can confirm that diminished specific antibody response is not due to the lack of germline variability [31, 32].

Previous works have identified three isotypes (kappa, sigma and lambda-2, alternatively iota-1, iota-2 and iota-4) for immunoglobulin light chains in the Atlantic cod [22, 33, 34]. We confirmed their presence, each located at separate chromosomal locations, exhibiting a clustered organization, but with notable variations. Most kappa chains possess inverted V genes, leading to inversions during recombination with J genes and potentially offering more avenues for secondary rearrangements. However, a smaller family of V genes are in the same orientation as the J and C, for which the classical recombination mechanism must be used. Notably, kappa chains have the largest number of clusters yet observed in fish. These clusters likely resulted from recent duplications, as evidenced by moderate receptor sequence variability and limited germline sequence variability and making it unlikely to be a compensatory mechanism for the lack of repertoire diversity. The conserved promoters and recombination signals across chains raises questions about the impact of the increased number of clusters on allelic exclusion, especially considering that instances of multiple light chain allele expression in a single B cell have been reported in rainbow trout [53].

Our work also established the basic characteristics of the repertoire by using deep sequencing technologies to target each isotype individually. Notably, by using shorter read sequencing, we focused on the analysis of CDR3 sequences in depth and assignment of used V genes using the forward and reverse reads respectively, instead of targeting the whole receptor sequence in a less comprehensive manner. In future works, using technologies with longer read lengths will be invaluable in the description of new alleles and the analysis of expressed CDR1 and CDR2 sequences. Saturation curves from our sequencing efforts suggested a comprehensive capture of diversity within our samples. While the total diversity of IgH chains likely surpasses our estimates, since only a part of the spleen was sampled, we believe to have captured the full receptor diversity for light chains, particularly sigma and lambda-2 chains. Interestingly, despite the extensive kappa chain cluster duplications, light chain diversity remains limited. This baseline established for light chain isotypes is critical for future studies exploring their specific functions.

An intriguing aspect of our findings is the differential impact of germline V-gene CDR3 length on receptor CDR3 length between heavy and light chains. In heavy chains, no clear correlation exists, and expressed CDR3 lengths form a bell-shaped distribution, with an average length of 13.26. In contrast to observations in torafugu [54], deletions, rather than insertions are favoured during the recombination process. Light chain receptor CDR3 length on the other hand appears to be predominantly determined by the germline IGLV CDR3 length, generating less variability during the recombination process. This is particularly noteworthy given the extensive cluster duplications of the kappa gene clusters, since that is typically considered a diversification mechanism. Both the favouring of deletions for heavy chains and the lack of insertions or deletions for light chains during recombination can be considered to further reduce expressed repertoire diversity and compound weak specific immune response. Our observations are consistent with the downregulation of TdT after expression of the heavy chain [55] and the size maintenance and contribution of non-germline nucleotides to sequence variability observed in mice [56], suggesting an interesting parallelism between teleost and murine BCR generation.

A significant overlap in repertoires across individual fish was observed, however, about 80% of receptors are unique to each fish analysed. This proportion, expected to decrease with more extensive sequencing, is still higher than seen in zebrafish [57] and torafugu [54], potentially indicating a less efficient antigen-driven selection in Atlantic cod, since the unchallenged repertoire should also be shaped by the shared environment of the fish. Our results confirm that all IGHV families are expressed in unchallenged fish [32], with several V genes being preferentially utilized in all individuals, despite not converging to identical amino acid sequences. The disproportionate use of IGHJ1 over IGHJ2 genes is more consistent than previously reported across V gene families. This hints at a potential functional distinction in immunoglobulins employing these J segments. Light chain V genes also exhibited preferential usage, albeit to a lesser extent. Notably, a striking overrepresentation of a single J gene in lambda-2 chains suggests a specific functional role.

In summary, the germline and expressed repertoire diversity for heavy chains in untreated Atlantic cod does not markedly differ from that in other fish species, to compensate or compound the species' limited ability to mount a specific immune response due to its unique immunogenetic architecture. On the other hand, extensive recent duplications of the kappa light chain genes do not yield a diverse repertoire raising the question of its origins and the evolutionary mechanism for maintaining such a high number of kappa chains. Future immune-challenge studies are anticipated to provide deeper insights into the temporal dynamics of repertoire structure. Nevertheless, our exhaustive cataloguing of genomic loci and deep sequencing of the unchallenged repertoire lays a foundational framework for an in-depth analysis of the specific immune responses in Atlantic cod on a molecular level and enhancing our understanding of fish immunology as a whole.

### Supplementary Information

Supplementary Material 1.

Supplementary Material 2.

Supplementary Material 3.

Supplementary Material 4.

Supplementary Material 5: Fig. S1. Phylogenetic tree of immunoglobulin heavy chain variable regions delineating V-gene families. Numbers indicate the bootstrap values from 1000 replicates on the neighbour-joining tree. Evolutionary distances were computed using the Kimura 2-parameter method and are in the units of the number of base substitutions per site. The rate variation among sites was modelled with a gamma distribution (shape parameter = 5).

Supplementary Material 6: Fig. S2. Phylogenetic tree of immunoglobulin light chain C genes. Neighbour-joining tree built from representative sequences from immunoglobulin light chain C-gene segments, with additional sequences from other fish species. Colours denote the major branches of light chains in accordance with isotypes. Numbers indicate the bootstrap values from 1000 replicates on the neighbour-joining tree. Evolutionary distances were computed using the Kimura 2-parameter method and are in the units of the number of base substitutions per site. The rate variation among sites was modelled with a gamma distribution (shape parameter = 5).

Supplementary Material 7: Fig. S3. Phylogenetic tree of immunoglobulin light chain V genes. Neighbour-joining tree of immunoglobulin light chain V gene phylogeny of representatives from identified genes in addition to V-gene segments from other fish. Colours correspond to the established isotypes of their corresponding C genes. Numbers indicate the bootstrap values from 1000 replicates on the neighbour-joining tree. Evolutionary distances were computed using the Kimura 2-parameter method and are in the units of the number of base substitutions per site. The rate variation among sites was modelled with a gamma distribution (shape parameter = 5).

Supplementary Material 8: Fig. S4. Shannon entropy indices for immunoglobulin germline gene segments. Yellow and grey fields represent CDR and framework regions, respectively. Letters on top denote the consensus sequence, dashes (-) are gaps, question marks (?) are too diverse to set consensus. Number in parentheses on top of each figure is the number of V and J genes in each group. A) Immunoglobulin heavy chain V3 family, B) Immunoglobulin kappa light chain V1 family and C) Immunoglobulin kappa light chain J3 gene segments. Other V- and J-gene families were not populous enough for entropy analysis.

Supplementary Material 9: Fig. S5. Number of unique receptors based on sequencing depth and abundance-based receptor rank and diversity for sigma and lambda-2 IgL chains. Number of unique receptors identified at the CDR3 amino-acid sequence level in relation to mapped sequencing reads for sigma (A) and lambda-2 (B) receptors. Subsamples of reads were created from mapped reads for each sample. Sampling depth was adjusted for each sample according to total number of mapped reads. Abundance-based receptor rank and proportion of repertoire of sigma and lambda-2 (D) light chains. Each point represents an individual receptor, stars denote D50 values for each sample. All axes are logarithmic except for lambda-2 light chains for better readability. D50 values and corresponding vertical lines were jittered slightly for better visibility.

Supplementary Material 10: Fig. S6. CDR3 length distribution of immunoglobulin sigma (A) and lambda-2 light chains (B). All samples were combined for calculating CDR3 length distributions.

Supplementary Material 11: Fig. S7. V gene usage in kappa immunoglobulin light chains. Genes are ordered based on their location in the genome. All samples were used in calculation of gene usage, error bars represent standard deviation between the different fish.

Supplementary Material 12: Fig. S8. V gene usage in sigma (A) and lambda-2 (B) immunoglobulin light chains. Genes are ordered based on their location in the genome. All samples were used in calculation of gene usage, error bars represent standard deviation between the different fish.

## Data Availability

Sequencing data will be available from EBI in the ArrayExpress collection in BioStudies, submission number E-MTAB-13762. https://www.ebi.ac.uk/biostudies/arrayexpress/studies/E-MTAB-13762
