# Supplementary figures and images for: Systematic characterization of immunoglobulin loci and deep sequencing of the expressed repertoire in the Atlantic cod (Gadus morhua)
Source: BMC Genomics. 2024 Jul 3;25:663. doi: 10.1186/s12864-024-10571-0 (PMC11223323; doi:10.1186/s12864-024-10571-0)

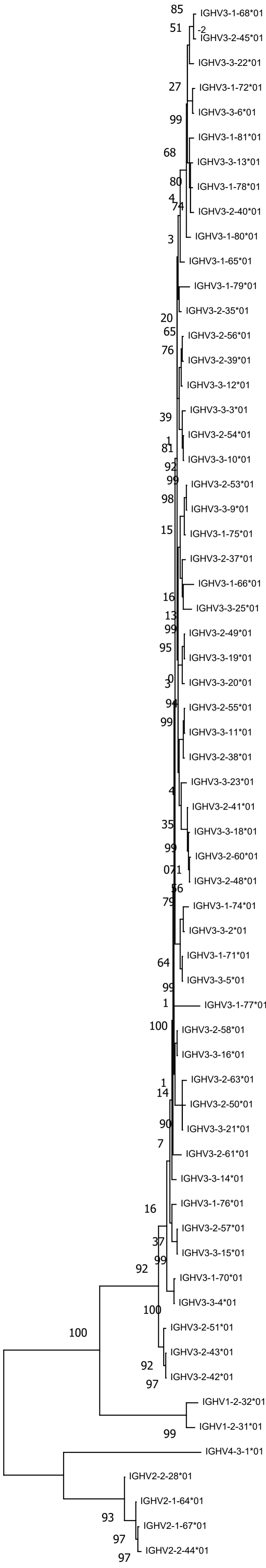

Supplement: Supplementary file 5 — Supplementary Material 5: Fig. S1. Phylogenetic tree of immunoglobulin heavy chain variable regions delineating V-gene families. Numbers indicate the bootstrap values from 1000 replicates on the neighbour-joining tree. Evolutionary distances were computed using the Kimura 2-parameter method and are in the units of the number of base substitutions per site. The rate variation among sites was modelled with a gamma distribution (shape parameter = 5). [file 12864_2024_10571_MOESM5_ESM.pdf]

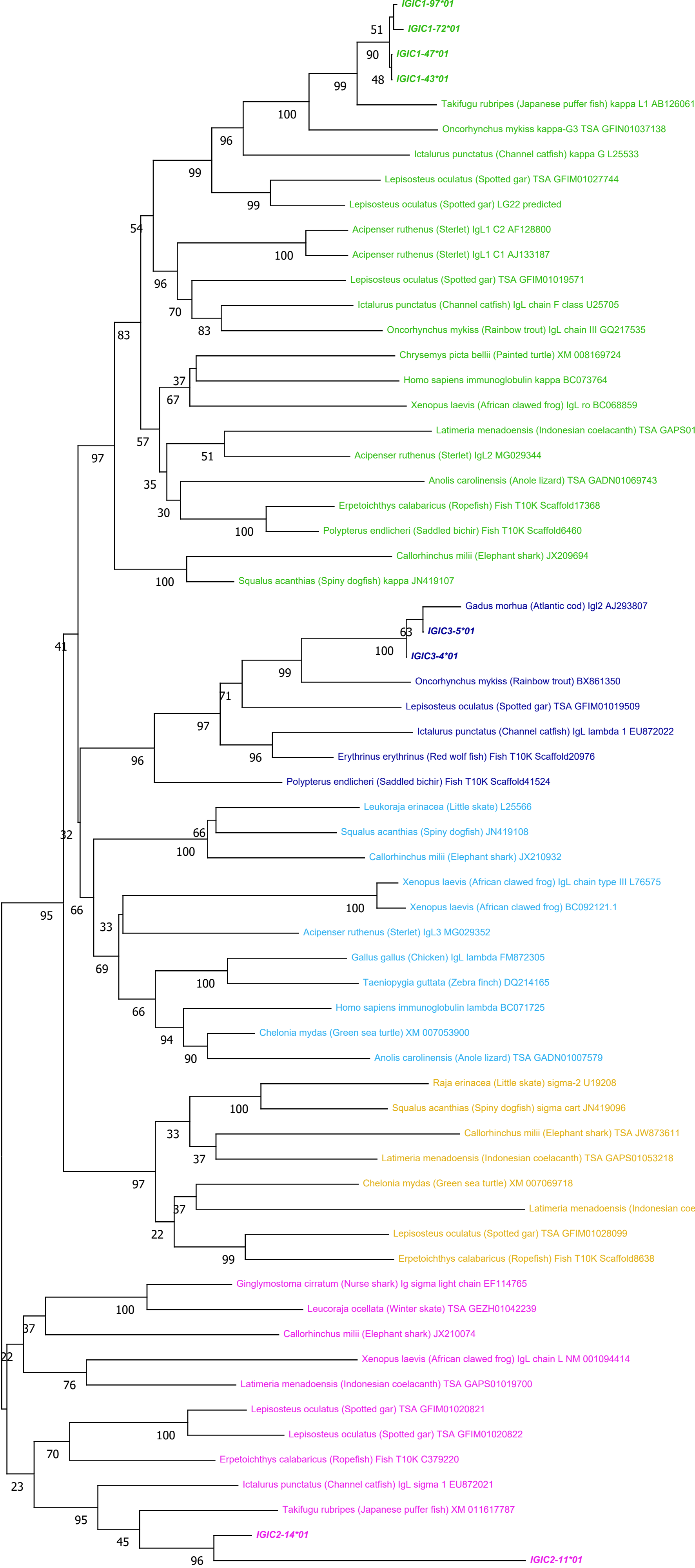

K

λ-2

λ

σ-2

σ

Supplement: Supplementary file 6 — Supplementary Material 6: Fig. S2. Phylogenetic tree of immunoglobulin light chain C genes. Neighbour-joining tree built from representative sequences from immunoglobulin light chain C-gene segments, with additional sequences from other fish species. Colours denote the major branches of light chains in accordance with isotypes. Numbers indicate the bootstrap values from 1000 replicates on the neighbour-joining tree. Evolutionary distances were computed using the Kimura 2-parameter method and are in the units of the number of base substitutions per site. The rate variation among sites was modelled with a gamma distribution (shape parameter = 5). [file 12864_2024_10571_MOESM6_ESM.pdf]

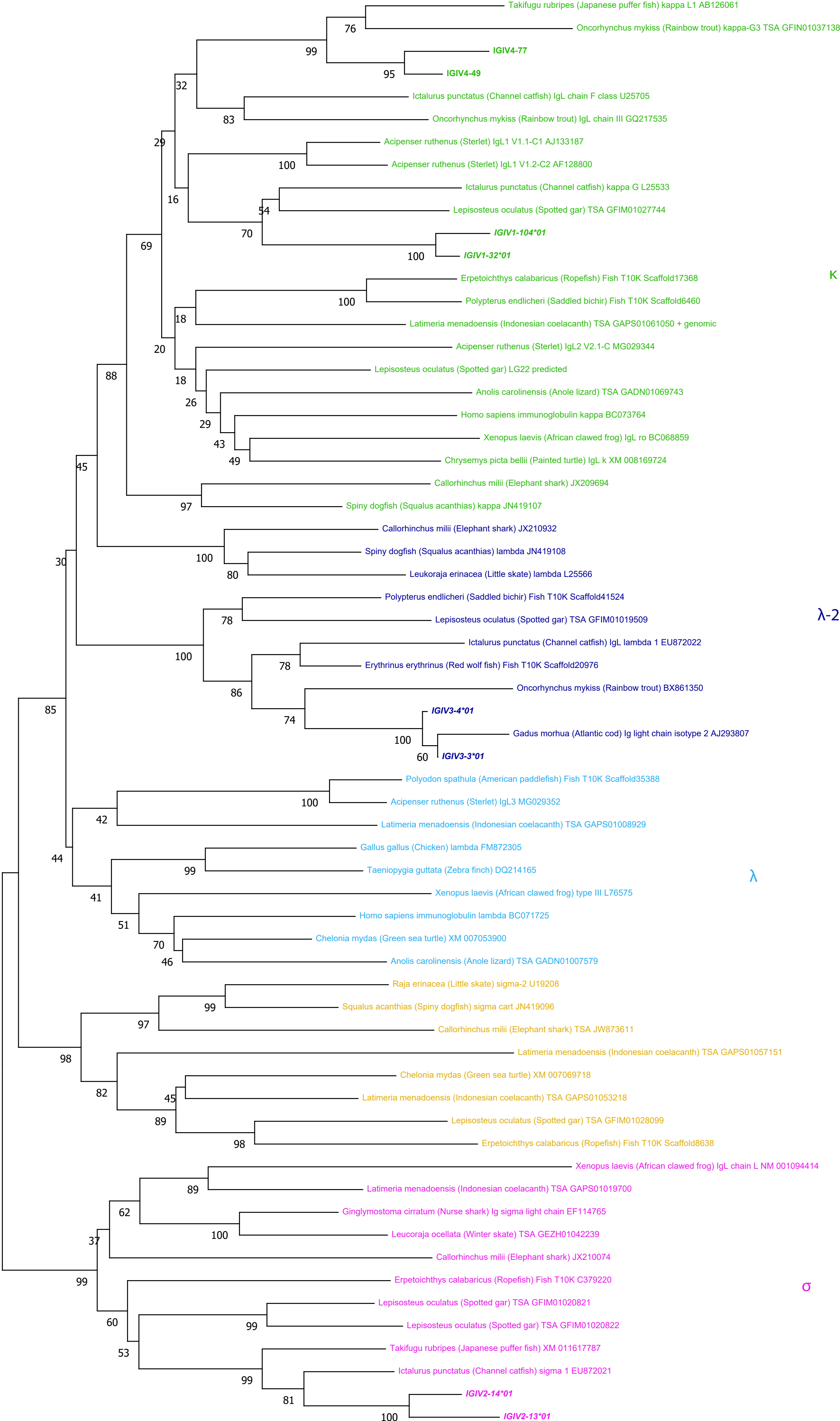

K

λ-2

λ

σ-2

σ

Supplement: Supplementary file 7 — Supplementary Material 7: Fig. S3. Phylogenetic tree of immunoglobulin light chain V genes. Neighbour-joining tree of immunoglobulin light chain V gene phylogeny of representatives from identified genes in addition to V-gene segments from other fish. Colours correspond to the established isotypes of their corresponding C genes. Numbers indicate the bootstrap values from 1000 replicates on the neighbour-joining tree. Evolutionary distances were computed using the Kimura 2-parameter method and are in the units of the number of base substitutions per site. The rate variation among sites was modelled with a gamma distribution (shape parameter = 5). [file 12864_2024_10571_MOESM7_ESM.pdf]

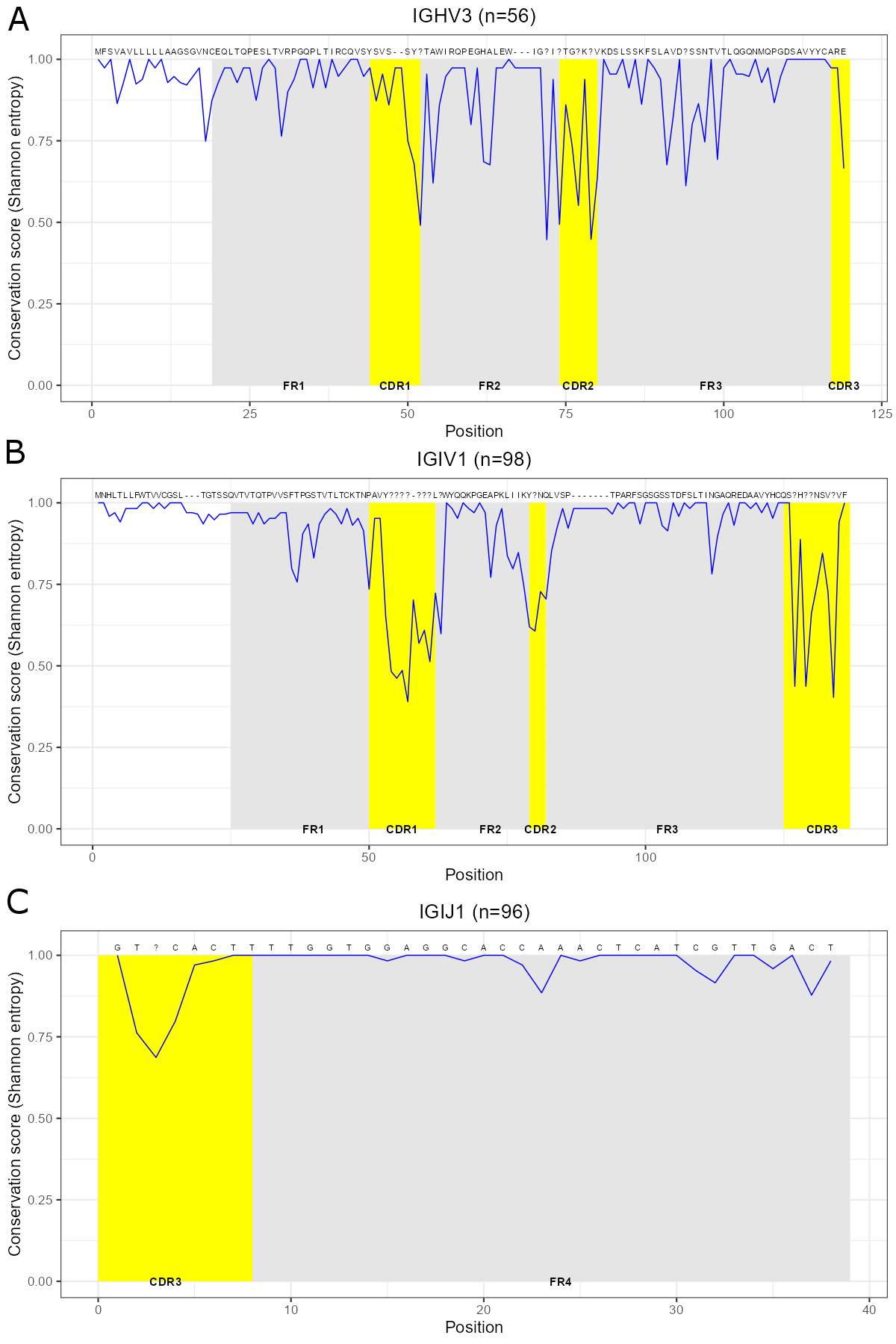

Supplement: Supplementary file 8 — Supplementary Material 8: Fig. S4. Shannon entropy indices for immunoglobulin germline gene segments. Yellow and grey fields represent CDR and framework regions, respectively. Letters on top denote the consensus sequence, dashes (-) are gaps, question marks (?) are too diverse to set consensus. Number in parentheses on top of each figure is the number of V and J genes in each group. A) Immunoglobulin heavy chain V3 family, B) Immunoglobulin kappa light chain V1 family and C) Immunoglobulin kappa light chain J3 gene segments. Other V- and J-gene families were not populous enough for entropy analysis. [file 12864_2024_10571_MOESM8_ESM.tiff]

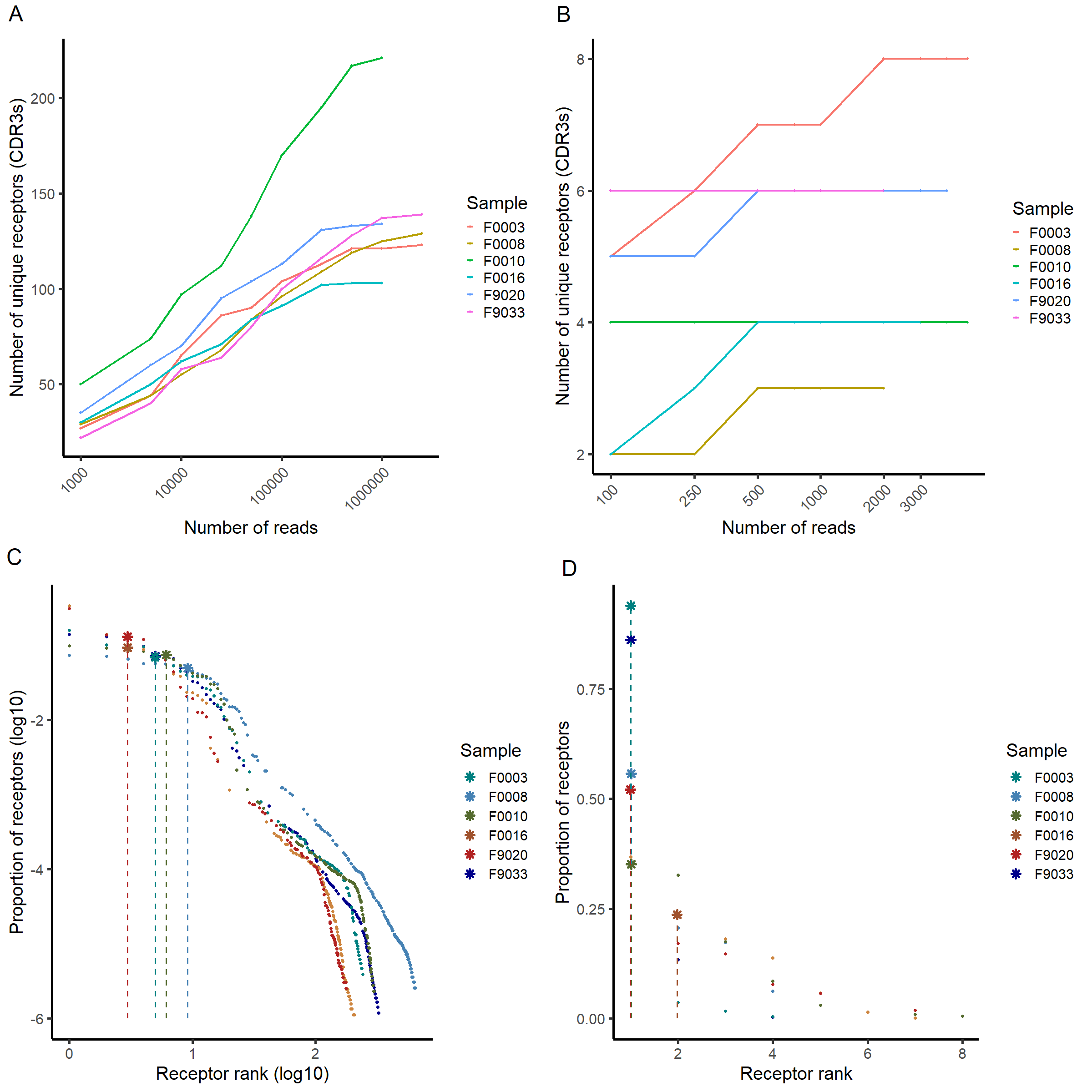

Supplement: Supplementary file 9 — Supplementary Material 9: Fig. S5. Number of unique receptors based on sequencing depth and abundance-based receptor rank and diversity for sigma and lambda-2 IgL chains. Number of unique receptors identified at the CDR3 amino-acid sequence level in relation to mapped sequencing reads for sigma (A) and lambda-2 (B) receptors. Subsamples of reads were created from mapped reads for each sample. Sampling depth was adjusted for each sample according to total number of mapped reads. Abundance-based receptor rank and proportion of repertoire of sigma and lambda-2 (D) light chains. Each point represents an individual receptor, stars denote D50 values for each sample. All axes are logarithmic except for lambda-2 light chains for better readability. D50 values and corresponding vertical lines were jittered slightly for better visibility. [file 12864_2024_10571_MOESM9_ESM.tiff]

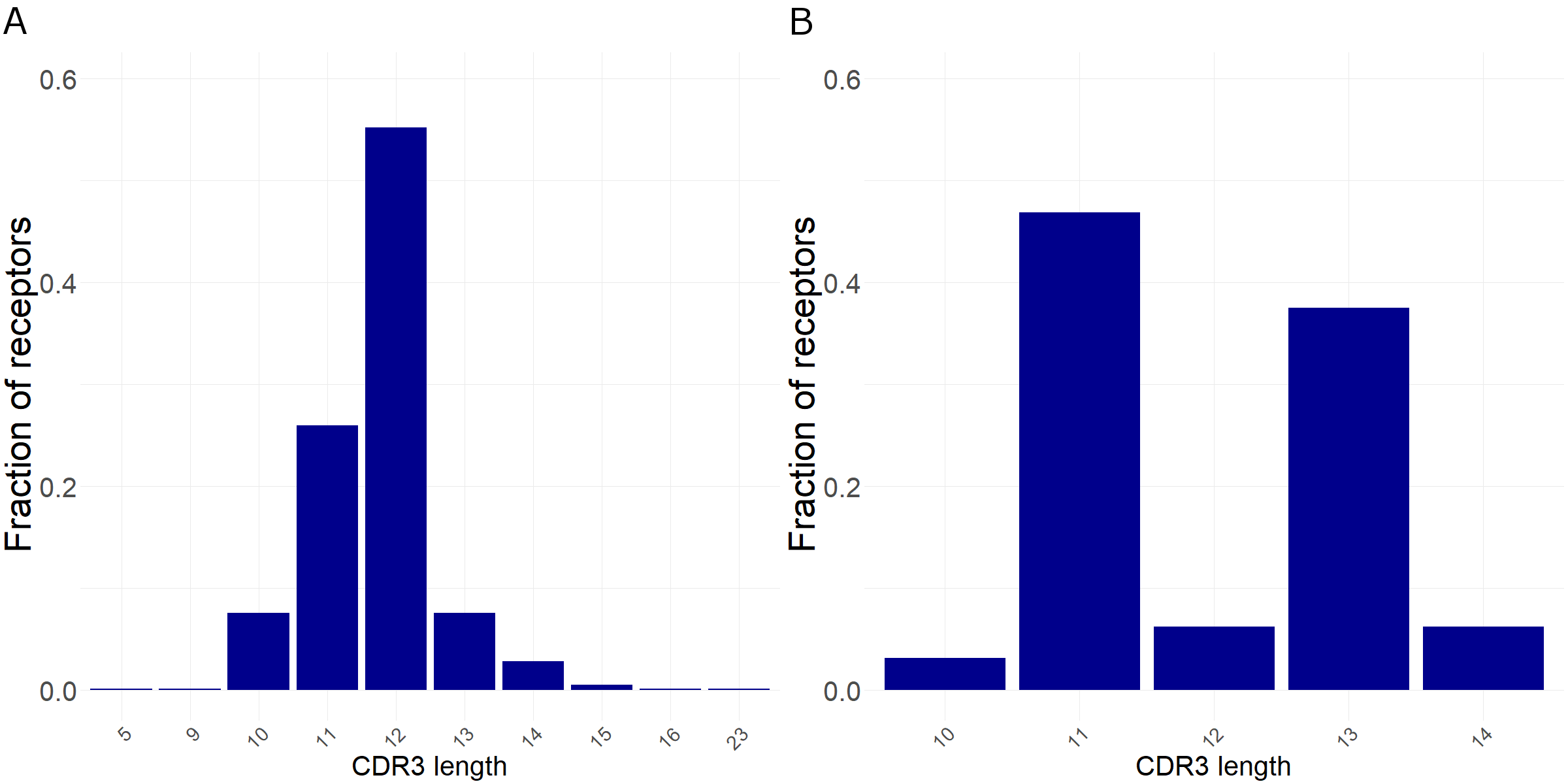

Supplement: Supplementary file 10 — Supplementary Material 10: Fig. S6. CDR3 length distribution of immunoglobulin sigma (A) and lambda-2 light chains (B). All samples were combined for calculating CDR3 length distributions. [file 12864_2024_10571_MOESM10_ESM.tiff]

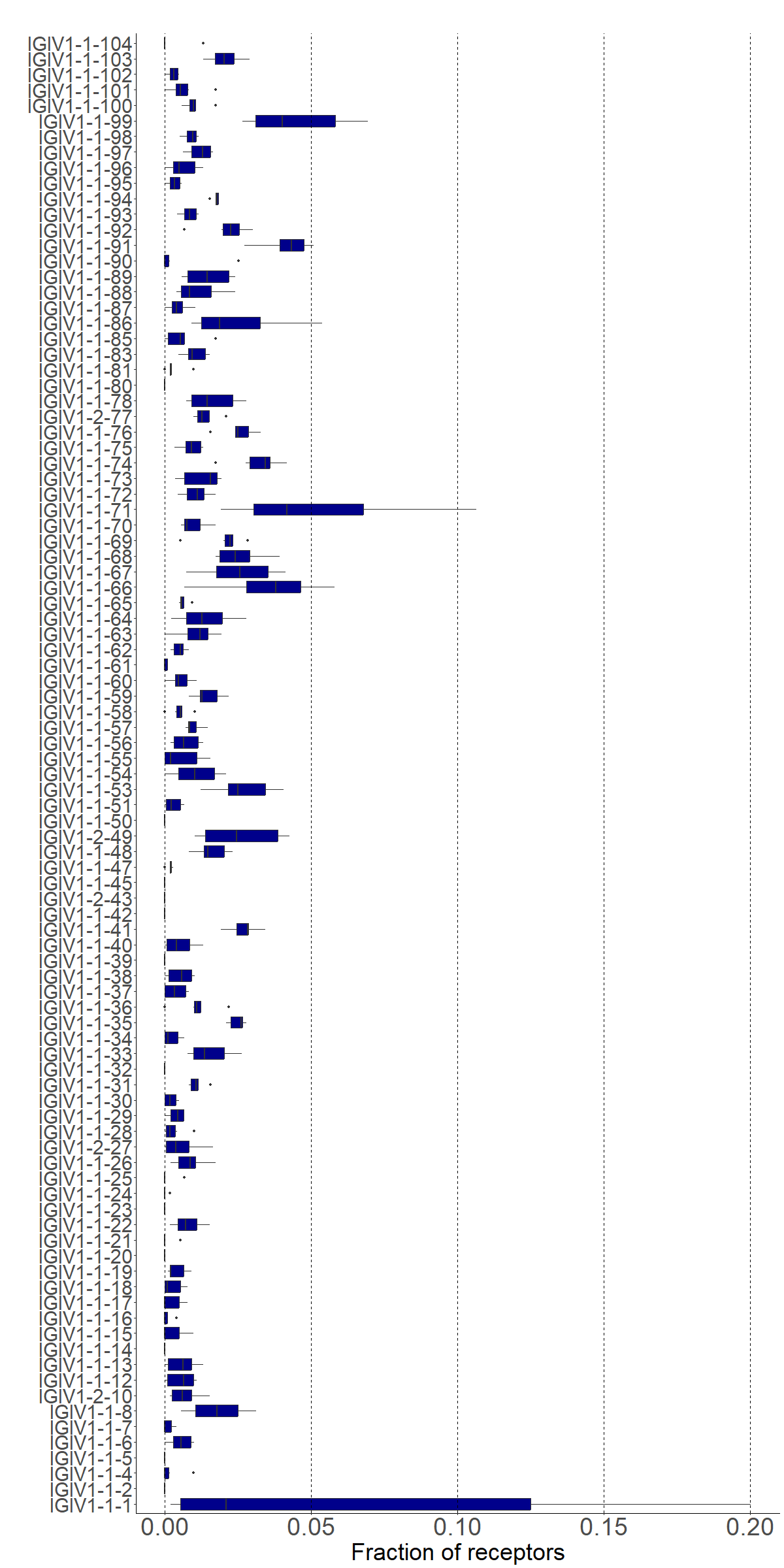

Supplement: Supplementary file 11 — Supplementary Material 11: Fig. S7. V gene usage in kappa immunoglobulin light chains. Genes are ordered based on their location in the genome. All samples were used in calculation of gene usage, error bars represent standard deviation between the different fish. [file 12864_2024_10571_MOESM11_ESM.tiff]

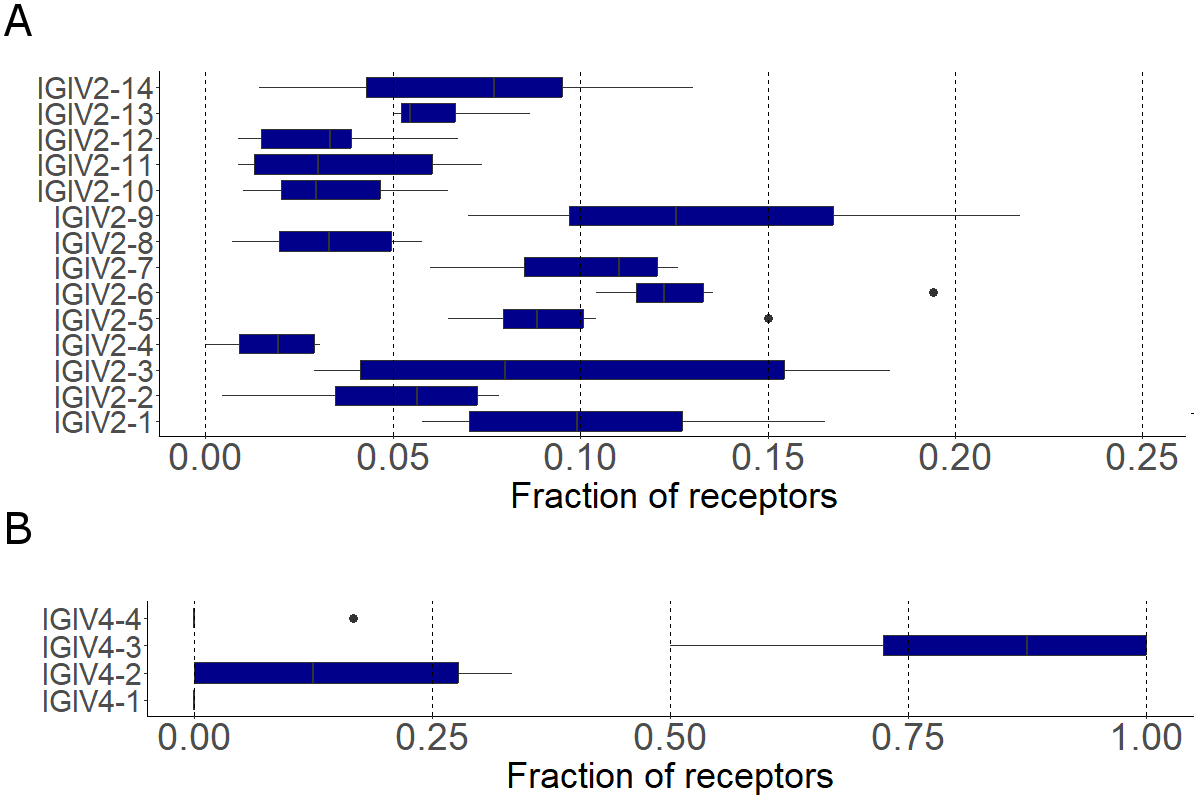

Supplement: Supplementary file 12 — Supplementary Material 12: Fig. S8. V gene usage in sigma (A) and lambda-2 (B) immunoglobulin light chains. Genes are ordered based on their location in the genome. All samples were used in calculation of gene usage, error bars represent standard deviation between the different fish. [file 12864_2024_10571_MOESM12_ESM.tiff]
